# Pheromone biosynthesis activating neuropeptide family in insects: a review

**DOI:** 10.3389/fendo.2023.1274750

**Published:** 2023-12-14

**Authors:** Xiaoyi Dou, Russell Jurenka

**Affiliations:** ^1^ Department of Entomology, University of Georgia, Athens, GA, United States; ^2^ Department of Plant Pathology, Entomology, Microbiology Iowa State University, Ames, IA, United States

**Keywords:** neuropeptides, PK/PBAN peptides, PK1/DH peptides, PK2/PBAN peptides, receptors

## Abstract

Neuropeptides are involved in almost all physiological activities of insects. Their classification is based on physiological function and the primary amino acid sequence. The pyrokinin (PK)/pheromone biosynthesis activating neuropeptides (PBAN) are one of the largest neuropeptide families in insects, with a conserved C-terminal domain of FXPRLamide. The peptide family is divided into two groups, PK1/diapause hormone (DH) with a WFGPRLa C-terminal ending and PK2/PBAN with FXPRLamide C-terminal ending. Since the development of cutting-edge technology, an increasing number of peptides have been sequenced primarily through genomic, transcriptomics, and proteomics, and their functions discovered using gene editing tools. In this review, we discussed newly discovered functions, and analyzed the distribution of genes encoding these peptides throughout different insect orders. In addition, the location of the peptides that were confirmed by PCR or immunocytochemistry is also described. A phylogenetic tree was constructed according to the sequences of the receptors of most insect orders. This review offers an understanding of the significance of this conserved peptide family in insects.

## 1 Introduction

Neuropeptides are very important in the physiology of insects. As the name indicates, they are peptides produced by the nervous system that act as endocrine and/or neuronal signals. They are synthesized in neurons and then travel down the axon to a synapse or can be released into the hemolymph for circulation through a neurohemal organ. Many neuropeptides have been identified from various insects and are involved in regulating physiology and behavior of insects (1, 2). Neuropeptides belong in families based on the primary amino acid sequence and physiological function. The major family of pyrokinin (PK)/pheromone biosynthesis activating neuropeptides (PBAN) is based on a conserved C-terminal motif of FXPRLamide which is the minimal requirement for biological activity (3, 4). The first PK was identified in the cockroach *Leucophaea maderae*, based on the stimulation of hindgut muscle contraction (5). The first PBAN peptide was isolated from the corn earworm moth, *Helicoverpa zea*, which regulates sex pheromone biosynthesis (4, 6). Since then, more peptides and their corresponding receptors have been identified through mass spectrometry, transcriptomics, proteomics, and genomics.

**Figure 1:**
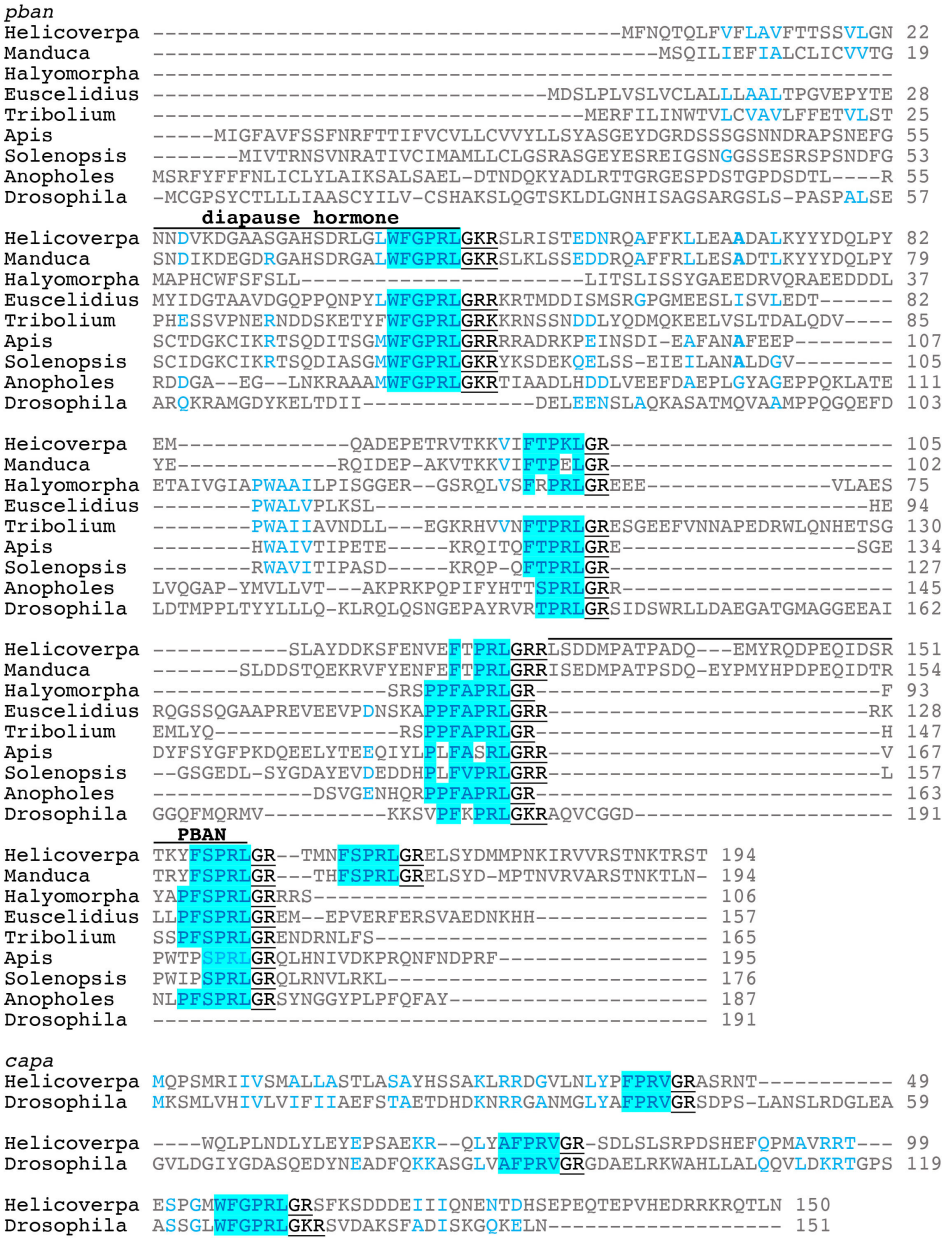
Representative gene sequence alignments for *pban* and *capa*. The diapause hormone and PBAN sequences as found in Lepidoptera are indicated on top of the sequences. The GK/R signal is underlined that produces the amide C-terminal ending. *Helicoverapa zea* and *Manduca sexta* are lepidopterans; *Halymorpha halys* and *Euscelidius variegatus* are hemipterans, Heteroptera and Auchenorrhyncha, respectively; *Tribolium castaneum* is a coleopteran; *Apis mellifera* and *Solenopsis invicta* are hymenopterans; *Anopholes gambiae* and *Drosophila melanogaster* are dipterans.

The gene encoding PBAN was first identified in *H. zea* (7). The *H. zea pban* gene can produce 5 neuropeptides including PBAN and diapause hormone (DH); the latter named because it induces embryonic diapause in the silkworm *Bombyx mori* (8). DH has also been referred to as trypto-PK due to the WFGPRLamide C-terminal ending (9). Here we will refer to DH- and PBAN-like peptides as PK1/DH and PK2/PBAN, respectively. The three other peptides have been named pyrokinins, subesophageal ganglion neuropeptides, or hugin peptides; the latter name is based on the *hugin* gene of *Drosophila melanogaster* (10). These peptides typically have a FXPRLamide C-terminal ending and will be referred to as PK2-1,2, and 3. Here we will align the neuropeptide gene sequences based on those of Lepidoptera where PK1/DH is the first peptide produced from the 5’ end followed by PK2-1 and 2, PBAN, and PK2-3 (**Figure 1**) (11). To date, the PK/PBAN family has been discovered in most insect orders and the function of these peptides has been characterized through different methods in many insects.

## 2 Function of the peptides

Previous research has demonstrated that the PK/PBAN family of peptides acts in a variety of functions, including sex pheromone biosynthesis in most moths (6, 12, 13) (but see 14, 15 for exceptions); melanization in moth larvae (16); embryonic diapause in *B. mori* (17); puparium formation in the higher dipteran, *Neobellieria bullata* (18); and breaking pupal diapause in heliothine moths (19, 20). More functions have been identified in recent years, indicating the pleiotropic nature of the peptide family. In Lepidoptera, several functions have been identified in addition to sex pheromone biosynthesis and embryonic diapause, including seasonal reproductive polyphenism in the tussock moth, *Orgyia thyellina* (21) and in addition to the breaking of pupal diapause in heliothine moths, the peptide could terminate larval diapause in *Omphisa fuscidentalis* (22). RNAi mediated knockdown of *pban* implicates other functions due to increased pupal mortality in *H. zea* (23) and delay of larval growth, interference of pupal development, and mortality in *H. zea* and *H. armigera* (24). In the fall armyworm, *Spodoptera frugiperda*, PBAN signaling regulates fecundity (25). Recently, the CRISPR/Cas9 gene editing tool was used to determine the role of *pban* in *Mythimna separata* (26). The study demonstrated that *pban* is necessary for density-dependent cuticular melanization during the late larval stages and is required for sex pheromone production in female adults.

Functions for the PK/PBAN family of peptides have been identified in other orders. In the fire ant, *Solenopsis invicta*, the knockdown of *pban* increases larval and adult mortality, and delays pupal development (23). It is also involved in production of the trail pheromone in the fire ant (23). DH-like peptides promote egg diapause in *Locusta migratoria* (27). The peptides produced by *hugin* are thought to be involved in regulating feeding behaviors in *D. melanogaster* (28). PKs were found to be involved in regulation of hindgut function in the adult mosquito, *Aedes aegypti* (29). Recently, PBAN from the western flower thrip, *Frankliniella occidentalis*, was shown to induce production of an aggregation pheromone (30).

## 3 PBAN-like gene encoding neuropeptides in insects

In most insects, two genes (*pban* and *capa*) encode several PK/PBAN-like peptides. The *pban* gene can encode up to five peptides, including a PBAN and a DH, was first identified in moths (7, 31). In *Drosophila*, the *hugin* gene is homologous to *pban* which encodes two PKs (10). The *capa* gene was first identified in *D. melanogaster*, encoding two periviscerokinin-like peptides with a PRV/Iamide and a PK with an WFGPRLamide C-terminal ending (32). Representative gene sequences for *pban* and *capa* are shown in **Figure 1**. Biological functions for the periviscerokinins include myotropic activation of muscles, including the heart, and control of diuretic action on the Malpighian tubules (33). So far, based on transcriptomics, the WFGPRLamide peptide produced by *capa* is found in almost all insects, with a few exceptions (11). It was not found in three species of Siphonaptera and only in the Symphyta of Hymenoptera. The *capa* of Apoidea and Formicidae could produce a peptide with a GYTPRLamide C-terminal ending in addition to the two periviscerokinins. This review will concentrate on the peptides produced by *pban* across Insecta (11).

**Figure 2:**
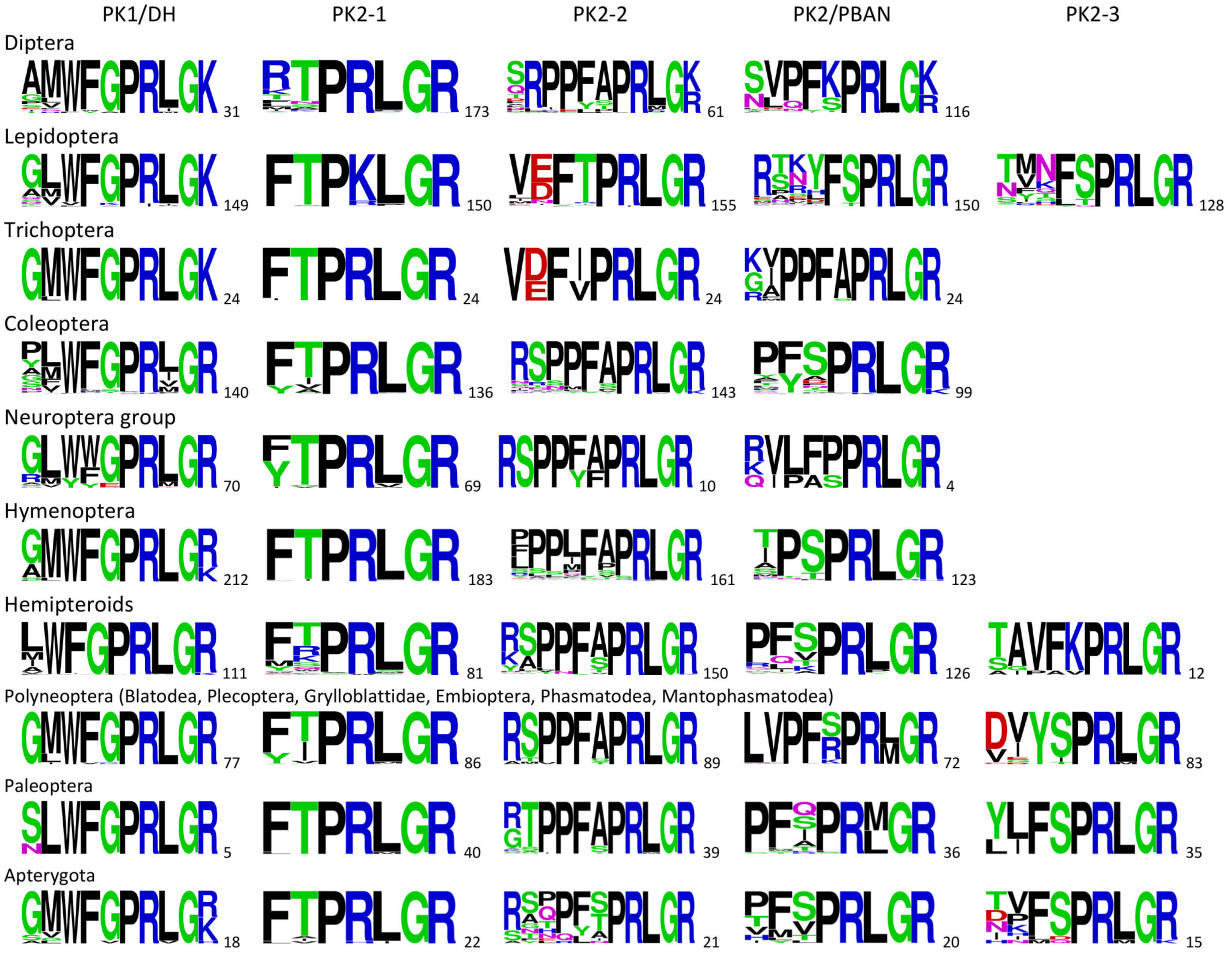
Consensus sequences of the C-terminal amino acids of peptides that could be produced by the *pban* gene of the indicated orders and groups of insects. All the peptides are C-terminal amidated using the glycine which precedes the R or K signal. The number of sequences used to generate the sequence logo is indicated as a subscript for each individual peptide. The sequence logo was created using Weblogo (34).

The number of transcriptome analysis of insect species has greatly increased in the past few years. This has greatly increased the number of species in which *pban* can be identified in NCBI databases and with increased numbers better inferences can be made. The peptides can be identified based on the GR or GK signal at the C-terminal end where the glycine is used to make the amide of the mature peptide (**Figure 1**). The N-terminal start of the peptide is also determined by a signal sequence that is more variable and also produces a variable length peptide. Here we will concentrate on the C-terminal ending in comparing *pban* across the Insecta as shown in **Figure 2**. A gene sequence does not necessarily mean that the insect will produce the mature peptide, which requires processing of the prepropeptide, but a transcriptome sequence does indicate that at least the mRNA is produced and thus the potential to produce a mature peptide.

It is immediately apparent that the PK1/DH peptide has the a consistent C-terminal ending and was found in almost all insects. The exception is the Hemiptera in which only the suborder Heteroptera consistently had PK1/DH and was more variable in the suborders Sternorryncha and Auchenorryncha. However, the Heteroptera were found not to have a PK1/DH sequence as was discovered in the brown marmorated stink bug (35). Also, in some Miridae (Heteroptera) *pban* produces only PK1/DH and PK2-1 with PK1/DH having an atypical FQPRSa C-terminal ending (36). PK2-3 was found in only a relatively few Hemiptera in the suborder Sternorrhyncha.

In the Apterygota, the most primitive hexapods include Diplura, Collembola, Archaeognatha, and Thysanura. All five peptides could be produced but PK1/DH was not found in 4 species of Diplura (37, 38). As described in Derst et al. (37), the Archaeognatha and Collembola have a gene that can produce both CAPA- and PK-like peptide sequences. The Paleoptera consisted of 35 species of Odonata and 5 species of Ephemeroptera, with the latter having the PK1/DH sequence. All species of Odonata lacked the PK1/DH sequence. The Polyneoptera included many hemimetabolous orders. The Polyneoptera order that is missing from **Figure 2** is the Orthoptera which apparently do not have a typical *pban*. All of the genes found in Orthoptera produce multiple copies of PK1/DH-like peptides that end in WFGPRXamide where X is variable but usually a valine. For example, in *Locusta migratoria*, five PK1/DH precursor genes were identified to produce five types of PK1/DH-like neuropeptides. A functional assay showed that four of the PK1/DH-like neuropeptides stimulated egg diapause under a short photoperiod, but no responses under a long photoperiod (27).

In the holometabolous insects, only the Lepidoptera have a *pban* that can produce all five peptides. The lower number of species in which PK2-3 was found in Lepidoptera is probably due to truncated sequences of transcriptome data rather than lack of a PK2-3 peptide. One notable difference in PK1/DH sequences is that the Tortricidae have a VFKPILa C-terminal ending (39). In the sister taxon to Lepidoptera, the *pban* of Trichoptera produced 4 peptides and all 24 species had a remarkable similar sequence structure.

The Neuroptera group included 42 species of Neuroptera, 17 species of Raphidoptera, and 11 species of Megaloptera. The gene structure of Neuroptera is different in that only the PK1/DH peptides were found. In addition, upstream of the PK1/DH sequence are two PRVamide sequences. These are similar to the perivicerokinins found in *capa* of other insects. Apparently, the peptides produced by *pban* and *capa* in other insects are consolidated together in one gene in Neuroptera. In Megaloptera and Raphidioptera, two *pban*-like genes were found; one similar to the Neuroptera and the other similar to other insects in that in addition to PK1/DH and PK2-1, PK2-2 could be produced. The PK2/PBAN sequence was found in only 4 species of Raphidoptera.

In Coleoptera, most species produced 3 peptides as illustrated in *Tribolium castaneum* (40). Notable exceptions include 4 species of Coccinellidae and 6 species of Lampyridae that lack the PK1/DH peptide sequence. The PK2-l and PK2-2 sequences were found in most Coleoptera. The corresponding PK2/PBAN sequence was found in fewer species but could be due to truncated sequences of transcriptomic data.

In Hymenoptera, all species had a very similar C-terminal sequence for the PK1/DH peptide. The remaining three peptides were not found in all Hymenoptera. Notably 8 species of the superfamily Chalcidoidea were found to lack the PK2-1 peptide. All 24 species of Vespidae only had the PK1/DH sequence and no other PK2 sequence.

In the lower Diptera (Nematocera), the PK1/DH sequence was found in Tipulidae, Chironomidae, and Culicidae. The PK1/DH sequence was lacking in all the other dipteran families including some lower Diptera like Psychodidae (41), Sciaridae and Keroplatidae. PK2-2 was found in the lower Diptera and in the lower Brachycera families. Only PK2-1 and PK2/PBAN-like sequences were found in the higher Brachycera families. Similar findings were found in a study by Farris (28).

## 4 PBAN location in CNS

The PK/PBAN family of peptides are detected in the brain, suboesophageal ganglion (SEG), certain cells of the corpora cardiaca, thoracic ganglia (TG), and abdominal ganglia (AG), based on immunohistochemistry using anti-PBAN in moths (42–44). The same sites were confirmed by RT-PCR in *H. armigera* (45). PVKs are typically found in the AG (46). In noctuid moths, the *pban* gene expression locations, the structure of PBAN precursors, and the processed neuropeptides are all very comparable. In other insects, such as *Drosophila* (47, 48), locusts (49), and cockroaches (50), similar expression profiles were found. Similar to other insects, PBAN immunoreactive neurons were detected in the fire ant central nervous system (CNS), but not in the last AG (51). The PK/PBAN-like peptides that could be released into the hemolymph were detected in the brain, SEG, TG, and AG of the mosquito nervous system by using immunohistochemistry (52). Products of the *capa* gene could be produced in the AG and the products of the *pban* gene could be produced in the SEG (52). This finding was supported by peptidomics studies in *A. aegypti* (53), *Delia radicum* (54), *Phlebotomus papatasi* (55) and *Lucilia cuprina* (56). In the stink bug, *Halyomorpha halys* (Hemiptera), the *capa* gene was highly expressed in the AG, whereas the *pban* gene was strongly expressed in the brain, specifically cerebral ganglia - gnathal ganglion (also known as the SEG) (35). The same results were found in silverfish, as in all Pterygota studied so far (38). It appears that in most insects *pban* is highly expressed in the SEG where PBAN could be released into circulation through the corpora cardiaca. Some of the PBAN containing neurons of the SEG also send axons down the ventral nerve cord to terminate in the last AG.

## 5 Receptors

Receptors for the PK/PBAN family of peptides are G-protein coupled receptors (GPCR) which are characterized by having seven transmembrane domains. The receptors (r) could be classified into two groups: PK2/PBANr and PK1/DHr based on whether they bind PK2/PBAN-like sequences or PK1/DH-like sequences. The PBAN/PK2r have been cloned and characterized in many insect species starting with *D. melanogaster* (57) and followed by *H. zea* (58) and *B. mori* (59) and later other moths (60). In addition, receptors have been detected in the transcriptome of pheromone glands from various female moths (61–65). In other insects the receptors have been identified in the mosquitoes *Anopholes gambiae* (66) and *Aedes aegypti* (67), and the beetle, *Tribolium castaneum* (40, 68).

The first PK1/DHrs were identified in *Drosophila* and confirmed using a PK1/DH peptide ligand (69), and then in *B. mori* (70). Their ligands are similar with a WFGPRLamide C-terminal ending. Previous research demonstrated that the WFGPRLamide peptide activated PK1/DHr in *Drosophila*, *An. gambiae*, and *B. mori* at low nM levels while the other PKs require significantly greater ligand concentrations (67, 69, 70). In *R. prolixus*, three PK1/DHr variants have been cloned, and they showed different dose responses to PK peptides (71). In *H. zea*, a PK1/DHr has been identified and has responses to both PK1/DH and PK2 peptides (72). A PKr was also identified in the southern cattle tick, *Rhipicephalus (Boophilus) microplus* (73) and was found to be basal to both the PK2/PBAN and PK1/DH receptors. The knockdown of the tick *pkr* caused an increase in mortality and decreased weight of both surviving females and subsequent egg masses indicating a function in reproduction (74).

**Figure 3:**
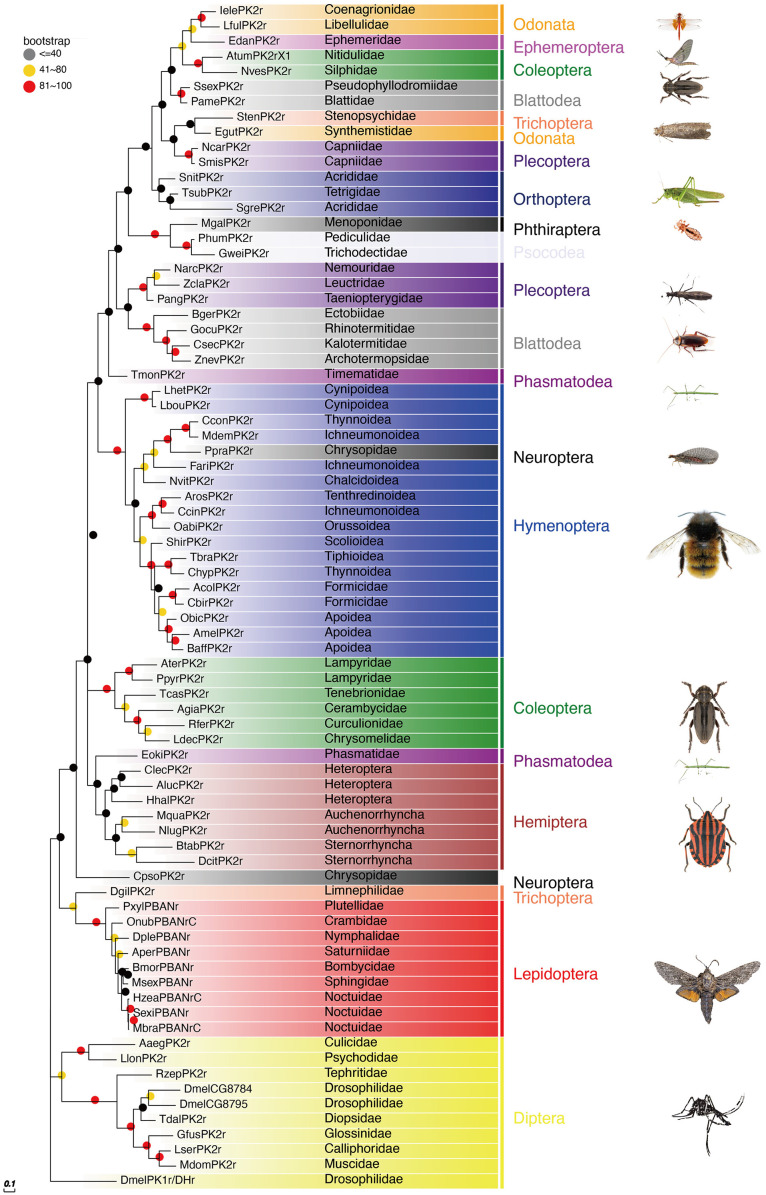
Phylogenetic relationship of PK2/PBANr representative sequences. C-terminal and N-terminal sequences of the receptors were removed then aligned using ClustalW, and the phylogeny calculated using the Maximum Likelihood algorithm with 100 bootstrap replicates. The family and order name are shown in the tree. The tree was modified using Evolview (75).

**Figure 4:**
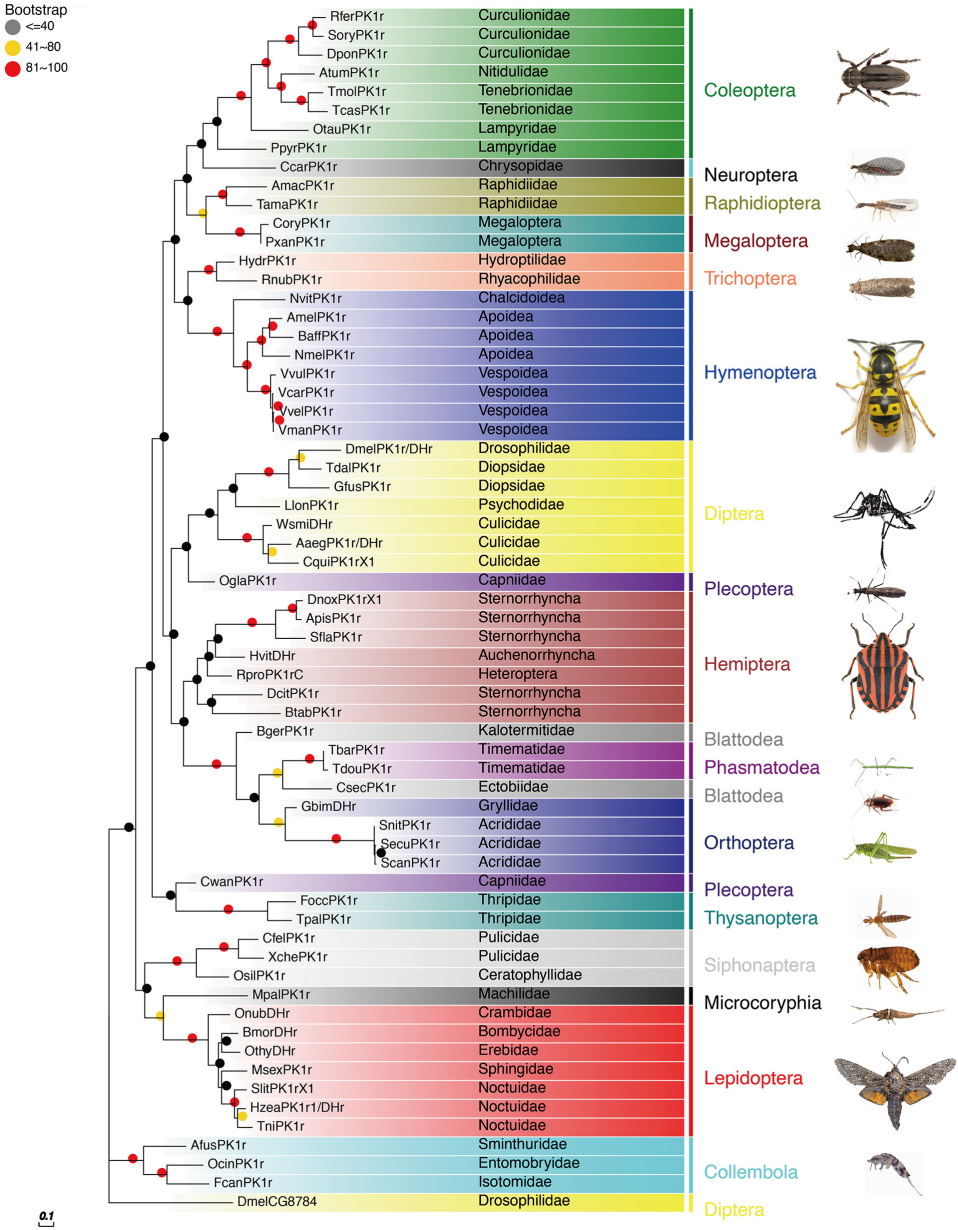
Phylogenetic relationship of PK1/DHr representative sequences. C-terminal and N-terminal sequences of the receptors were removed then aligned using ClustalW, and the phylogeny calculated using the Maximum Likelihood algorithm with 100 bootstrap replicates. The family and order name are shown in the tree. The tree was modified using Evolview (75).

The PBAN receptors have been detected in most insect orders. The phylogenetic tree shown in **Figure 3** for PK2/PBANr was built with representative sequences from 19 orders and in **Figure 4** for PK1/DHr was built with representative sequences from 18 orders. The sequences were selected using BLAST results to non-redundant protein sequences with known sequences from *D. melanogaster*, *H. zea*, *A. mellifera*, *B. mori*, and *T. castaneum*, and the remaining receptors were detected in the transcriptome shotgun assembly database but without annotation. As demonstrated in previous studies the PK2/PBANr and PK1/DHr are closely related but distinguished from each other in a phylogenetic tree (11, 57, 69). The PK2r and PBANr are grouped together since they have similar ligands with a FXPRLamide C-terminal ending, while PK1r and DHr are grouped together as they are activated by similar ligands with a WFGPRLamide C-terminal ending.

Although representatives of most insect orders have a PK2/PBANr there are some families that do not have these receptors. One such family is the Vespidae in the Hymenoptera. This corresponds to the finding that all of the Vespidae have a *pban* that only has the PK1/DH sequence and does not have the PK2 sequences.

Due to the importance of PBAN in regulating sex pheromone biosynthesis in Lepidoptera, the PBANr has been extensively studied in moths. The first PBAN receptor was identified in *H. zea* (58) and followed by in *B. mori* (59). PBAN binds to its receptor, triggering a signal cascade that stimulates the sex pheromone biosynthetic pathway by activating calcium/calmodulin-dependent adenylate cyclase and cyclic AMP (cAMP) as shown in heliothine moths. The cAMP then activates acetyl-CoA carboxylase to initiate sex pheromone biosynthesis (76). Whereas, the PBAN signal promotes fatty acid removal from lipid droplets and activates fatty acid reductases in *B. mori* (76, 77).

PBAN has a relatively low threshold for activating the receptor. PBANr in *H. zea* could be activated at EC_50_ of 25 nM (58). Interestingly, PBAN activates PBANr from *A. pernyi* in CHO-K1 and HEK293 cells at two different concentrations (2.32 nM and 28.6 nM, respectively). However, the activation of PBANr does not activate the cAMP elevation pathway when expressed in the mammalian cell line HEK293 (78). Several variants from one to three were found in Lepidoptera (79). All three receptor sequences were identical except for C-terminal extensions. Only one PK/PBANr was detected in *S. littoralis* and it responded to both PBAN and DH with EC_50_ at 0.12 pM and 0.013 pM, respectively (80). In *H. peltigera*, the PBANr responses to PBAN at a similar concentration but was not activated by DH (80). Two PBANr variants were detected in the transcriptome of *H. zea* (61) but only one was confirmed by functional analysis (58). In *M. brassicae*, three PBANr variants were identified and all showed activity in inducing Ca^2+^, but only PBANr-B and PBANr-C caused ligand-induced internalization when expressed in Sf9 cells (81). In *O. nubilalis*, three PBANr variants and one DHr were detected in pheromone glands, but only variant C was activated by PBAN with an EC_50_ of 44 nM and variants A and B were inactive. The DHr was activated by DH with EC_50_ at 150 nM (82). Interestingly, PBANr was also detected in male *H. armigera* hair-pencil-aedaegus complexes. The knockdown of this receptor significantly reduced the amount of various male components including free fatty-acid components and alcohol components by 58%–74% (83). These results indicate that PBAN could be involved in regulating male hair-pencil components that are involved in courtship behaviors.

Studies using PKr from *Drosophila* and *An. gambiae* have revealed similar cross-reactivity of ligands across the PK1/DH and PK2/PBAN receptors, despite the fact that the receptors responded differently to PK1/DH and PK2/PBAN peptides (57, 66). Additionally, neuromedin U can activate the *H. zea* PBANr but not the *D. melanogaster* PK1r (84), indicating that these two receptors can be distinguished from one another. These findings show that the PK/PBANr can be activated by a variety of peptides with a PRLamide ending.

## 6 Conclusion

PK/PBAN family peptide sequences have been extensively investigated by genomics, transcriptome, and peptidomics, and the neuronal sites are examined by immunocytochemistry, peptidomics, and PCR. RNA interference (RNAi) and gene editing are also used to examine the function of these peptides. It is important to highlight that most insect orders include the genes encoding these peptides and their corresponding receptors. Some insect orders have conserved functions, however, the functions in many insects remain unknown. In this review, we analyzed the function of the peptides in different orders, several recent functions have been identified, such as cuticular melanization in the oriental armyworm larvae (26) and aggregation pheromone production in the western flower thrip (30), etc. Also, we analyzed the genes encoding these peptides. In most insects, the *pban* gene produced multiple peptides, including PBAN, DH, and several PK-like neuropeptides, while the *capa* gene produced one PK1/DH and multiple PVKs. Even though some receptors exhibit cross-reactivity to several ligands, these various C-terminal ending peptides are the ligands for activating their particular receptors. Since the importance of the PK/PBAN family peptides and the conserved nature across insects, it is important to integrate the molecular modeling and biochemical studies to make contribution to insect control strategies. The design of the antagonist to FXPRLa peptides and receptors has the potential application for pest management (85).

## Author contributions

RJ: Writing – review & editing. XD: Writing – original draft.
